# *Bifidobacterium longum* R0175 attenuates post-myocardial infarction depressive-like behaviour in rats

**DOI:** 10.1371/journal.pone.0215101

**Published:** 2019-04-22

**Authors:** François Trudeau, Kim Gilbert, Annie Tremblay, Thomas A. Tompkins, Roger Godbout, Guy Rousseau

**Affiliations:** 1 CIUSSS du Nord-de-l’Île-de-Montréal, Hôpital Sacré-Cœur, Montréal, Canada; 2 Lallemand Health Solutions, Montréal, Canada; 3 Department of Psychiatry, Université de Montréal, Montréal, Canada; 4 Department of Pharmacology and Physiology, Université de Montréal, Montréal, Canada; University of PECS Medical School, HUNGARY

## Abstract

Caspase-3 activation in the limbic system and depressive-like symptoms are observed after an acute myocardial infarction (MI) and studies suggest that inflammation may play a significant role. Combined treatment with the probiotic strains *Bifidobacterium longum* and *Lactobacillus helveticus* in rats has been shown to attenuate caspase-3 activation and depressive-like behaviour together with a reduction in pro-inflammatory cytokines. The present study was designed to determine the respective contribution of these two strains on caspase-3 activity in the limbic system and on depressive-like behaviour. Sprague-Dawley rats were assigned to one of four groups: Vehicle, *L*. *helveticus* R0052, *B*. *longum* R0175 and *L*. *salivarius* HA-118, administered orally for 14 days (10^9^CFU daily) before inducing MI by occlusion of the left anterior descending artery for 40 min followed by 14 days of reperfusion. Animals were then tested for socialisation, passive avoidance and forced swim test to assess depressive-like behaviour. At day 18 the animals were sacrificed; infarct size was estimated, plasma C-reactive protein concentration and brain caspase-3 activity were measured. Results indicated that infarct size did not vary across the different treatments. Rats treated with *B*. *longum* spent more time socializing, learned more rapidly the passive avoidance test and spent less time immobile in the forced swim test compared to the vehicle groups. Caspase-3 activity and plasma C-reactive protein concentrations were reduced in the lateral and medial amygdala as well as in the dentate gyrus of *B*. *longum*-supplemented animals. The only significant effect in the two groups receiving *Lactobacilli* compared to vehicle was that rats receiving *L*. *salivarius* learned more rapidly in the step-down passive avoidance test.

In conclusion, most of the beneficial effects that we previously reported with the combination of two probiotic strains in our experimentation regarding post-myocardial infarction depression are related to *Bifidobacterium longum*.

## Introduction

Myocardial infarction (MI) is an inflammatory disease that has several impacts on the health of surviving patients [1]. One of the consequences that is regularly observed following myocardial infarction is the development of depression [2] which increases the mortality rate of these patients over the months that follow [3]. Although the mechanism linking the cardiac tissue and the brain has not been fully elucidated, inflammation is certainly a key component in this relationship [4]. In experimental studies, interventions mitigating the inflammatory process following myocardial infarction have been shown to attenuate depressive-like behaviour. Pentoxifylline [4], omega-3 fatty acids [5]and resolvin D1 (RvD1) [6] are among the experimental interventions that have been shown to reduce post-MI depressive-like behaviour as well as having an effect on inflammation.

Administration of a probiotic formulation containing *Bifidobacterium longum* R0175 and *Lactobacillus helveticus* R0052 also significantly improves the behaviour of post-MI animals [7]. In a rat model of post-MI depression-like behaviour, administration of this formulation significantly reduced the time of immobility in the forced-swim test and the time to learn the passive avoidance step-down test, and increased the duration of interaction between congeners. Similarly, it has been observed by Li et al. that a multi-strain probiotic formulation, including *B*. *longum* R0175 and *L*. *helvetic*us R0052, attenuated the chronic mild stress induced anxiety and depressive-like behaviours [8].

The activity of caspase-3, an enzyme involved in apoptosis, was increased in the limbic system following MI [9], an effect that was mitigated by a probiotic combination formulation [10]. This effect was contemporaneous to a reduction in the level of circulating pro-inflammatory cytokines, such as CCL-2 [5].

*B*. *longum* is a member of the *Bifidobacterium* genus known as fermentative bacteria. It uses carbohydrate to form acetic and lactic acids. Among the different effects reported for *Bifidobacteria* is a reduction of inflammation caused mainly by a reduction of pro-inflammatory cytokines [11, 12], which could be beneficial to prevent the development of post-MI depression.

*L*. *helveticus* is a member of the *Lactobacillus* genus, which are gram-positive and involved in the fermentation of lactose. Many studies have reported that *Lactobacilli* administration has positive effects on stress and depression [13, 14] through a mechanism that is still undefined. Both probiotics could be beneficial to attenuate post-MI depressive-like symptoms, but their individual contributions are, as yet, unknown.

The present study has been designed to determine whether individual strains (*B*. *longum* R0175, *L*. *helveticus* R0052) could have positive effects on post-MI behaviour and the activity of caspase-3 in different regions of the limbic system. In addition to these two strains, *L*. *salivarius* HA-118, for which no anti-depressant effect was observed [15], was also tested in the present model to assess for strain-specificity of the effects.

## Materials and methods

### Ethics statement

The experiments complied with the animal care guidelines published by the Canadian Council on Animal Care, and the procedures performed were approved by the local Animal Care Committee of the Hôpital du Sacré-Coeur de Montréal.

### Experimental design (Table 1)

**Table 1:** Sequence and duration of interventions and assessments periods during the entire experimental protocol.

Acclimatisation after arrival: 3 days	Treatment period: 14 days	Acute Myocardial Infarction	Reperfusion period: 14 days	Behavioural testing: 4 days	Sacrifice
Days -2 to 0	Days 1–14	Day 14	Days 14–28	Days 28–32	Day 32

Forty male Sprague-Dawley rats from Charles River Canada (Saint-Constant, Quebec, Canada) were used. They were housed in a controlled experimental environment (20–25°C, 40–50% humidity, 12 h light per day, lights on at 8:00 am) for three days without intervention to allow them to acclimatise. Then, the rats were separated into four groups according to the strains of bacteria to which they were subsequently exposed: vehicle (maltodextrin); *Lactobacillus helveticus* R0052; *Bifidobacterium longum* R0175; *Lactobacillus salivarius* HA-118. The rats had access to water (containing 10^9^ colonising bacterial units or CFU) renewed and weighed daily, from which they drank between 40 mL and 60 mL per day. The rats in each group were maintained under these experimental conditions for 14 days, after which MI was induced by standardised occlusion of the left anterior coronary artery (LAD) for 40 min. The rats were then kept under the same conditions as before MI (environment, standard diet, specific water, daily weighing). Finally, 14 days after the infarction, the rats were subjected to behavioural tests over a 4-day period (passive avoidance, social interaction, and forced swimming), each aiming to assess typical depressive-like behaviours in rats[16]. Finally, the rats were sacrificed to collect blood, brain and heart samples on which analyses were performed (infarct size and quantification of apoptotic protein activity).

### Probiotics

*L*. *helveticus* R0052, *B*. *longum* R0175 or L. *salivarius* HA-118 were administered by dissolving the freeze-dried culture or the vehicle only (maltodextrin) in 200 mL of tap water. Each rat in the probiotic groups received a daily dose of 10^9^ colony-forming units. The drinking solution was freshly prepared each day for the duration of the experiments. Water intake was monitored throughout the entire investigation to ensure sufficient bacteria were administered.

### Surgical procedures

Anesthesia was induced by a solution of ketamine and xylazine (80 mg/kg and 10 mg/kg i.p., respectively) and maintained with isoflurane (1%). The animals were intubated and placed on a respirator. Following left thoracotomy, the left anterior descending coronary artery was occluded with a 4.0 silk suture and plastic snare. Ischemia was assessed with ST segment alterations and ventricular subepicardial cyanosis. The suture was removed after 40 min of ischemia to begin the reperfusion. The thorax was then closed and the rats were injected with analgesics (2 mg/kg of butorphanol) [17]. Animals were sacrificed by decapitation 18 days following reperfusion to prevent biochemical pathway modifications.

### Infarct size measurement

The hearts were removed immediately after euthanasia and placed in dishes kept on crushed ice. They were washed with saline by retrograde perfusion via the aorta. The left anterior descending coronary artery was occluded at the same site as for MI induction to map the area at risk (AR) by Evans blue infusion (0.5%). The hearts were frozen (-80°C for 5 min), sliced into 4 transverse 2 mm sections and placed in 2,3,5-triphenyltetrazolium chloride solution (1%, pH 7.4) at 37°C for 10 min to better distinguish the area of necrosis (I) from the AR. The different regions were carefully drawn on a glass plate, photocopied, and cut. Thereafter, the complete infarct region, AR and left ventricle (LV), were weighed separately to express MI as percentages of necrosis (I) of the AR (I/AR x 100), and AR as percentages of the LV area (AR/LV x 100).

### Biochemical analyses

#### Caspase-3 activity

Caspase-3 activity was measured according to the protocol described previously [17]. Tissues were homogenised by sonication in lysis buffer and incubated for 30 min on ice. The tissue homogenates were centrifuged at 4°C for 10 min. The enzymatic reactions, each containing 1X reaction buffer with 25 mg of protein (attested by the Bradford method) and fluorescent substrate (Ac-DEVD-AMC) (40 μM), were incubated in the dark for 3 h at 37°C. Reactions were stopped by the addition of 0.4 M NaOH and 0.4 M glycine buffer. Fluorescence was quantified by spectrofluorometry (Photon Technology International, Lawrenceville, NJ, USA) at an excitation wavelength of 365 nm and an emission wavelength of 465 nm.

### Behavioural measures

The tests used were selected based on their validity regarding depression-like behaviours. All tests were conducted individually, in the morning, starting 14 days after MI. The animals were subjected to one test per day.

### Social interaction test

Pairs of rats were placed in a clean shoebox for 10 min. During this period, two observers, without knowledge of the experimental condition, observed one animal each; the duration and number of interactions with the other rat were measured. Rats were tested between 9:00 a.m. and 11:00 a.m.

### Forced swim test

Rats were placed individually in a transparent 25 cm diameter pool filled with 30 cm of 22°C– 25°C water, with no possible escape. Two observers, without knowledge of the experimental condition, used identical stopwatches to time the immobile, swim, and escape trial duration for each animal. The test was conducted over two days; on day 1 rats were allowed 15 min of habituation, and the actual 5 min test was taken on day 2. Rats were tested between 9:00 a.m. and 11:00 a.m.

### Passive avoidance step-down test

Rats were placed individually in a test chamber (14 cm x 23 cm) on a Plexiglas platform (14 cm x 19 cm) flanked by an electrifiable grid (14 cm x 14 cm) located 2.5 cm lower than the Plexiglas platform. Upon placing four feet on the electrifiable grid, the animal received a mild, brief shock (5 mA for 1 s) and was removed from the test chamber. After 30 s, it was placed on the platform again. If the rat remained on the platform without going onto the grid for one min, it was removed from the test box for 30 s. The criterion was reached when the rat avoided going onto the grid for three consecutive trials. The number of trials needed to reach the test criterion and the time needed to learn the test were noted. Rats were tested the day before being sacrificed.

### Statistical analyses

The results are expressed as the mean ± SEM. Infarct size, AR, behavioural tests and caspase-3 activity were tested using one-way ANOVA, followed by Dunnett’s test post hoc when significant. The vehicle group served as the control. When variances were heterogeneous, a Brown-Forsythe correction was applied to determine if the ANOVA was significant. Plasma CRP concentrations were not normally distributed and were analysed using the Kruskal-Wallis test followed by the Mann-Whitney U test with a *p*-level adjusted for the number of comparisons with a correction of Bonferroni. All statistical analyses were performed using the SPSS software v. 24, with *p* < 0.05 as the significance threshold.

## Results

### Infarct size

**Fig 1:**
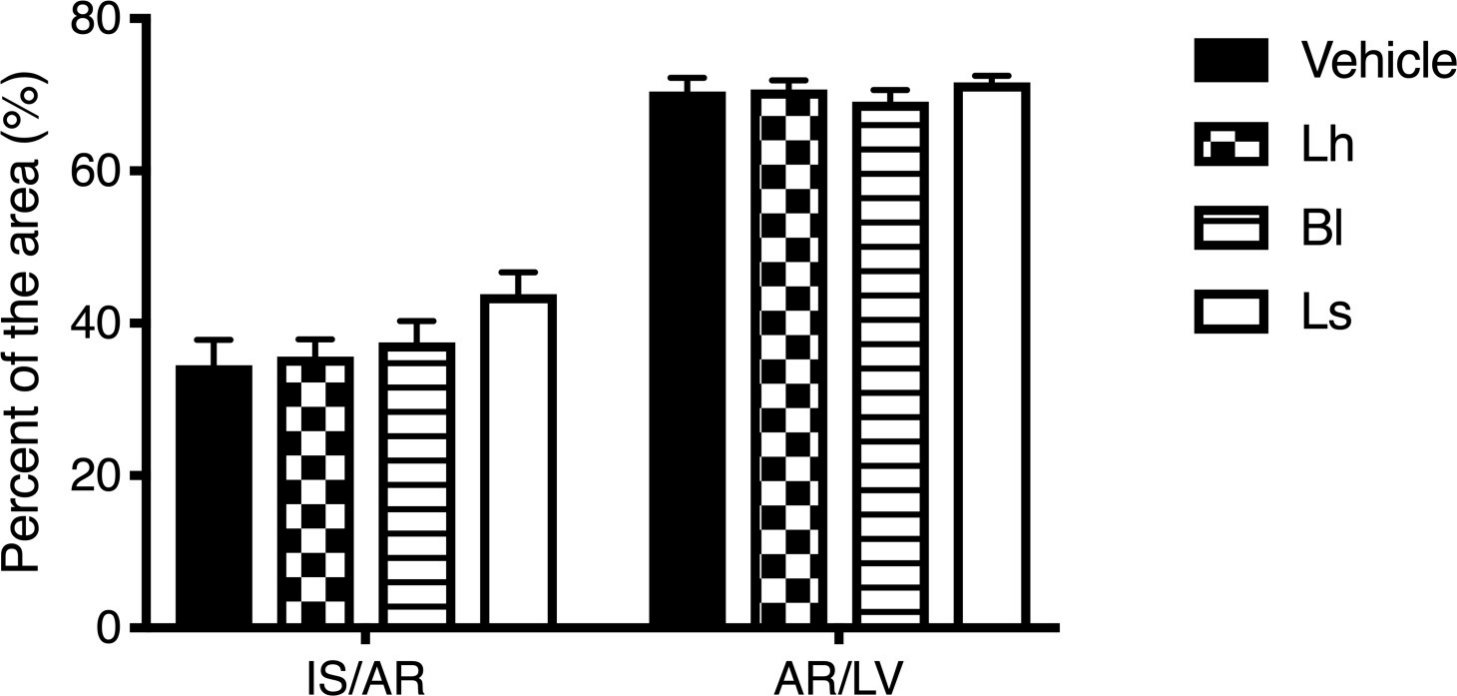
Infarct size (IS) expressed as a percentage of the area at risk (AR), and AR as a percentage of the left ventricle (LV) after a 24 h reperfusion. Note: *n* = 8–9 rats per group.

No mortality occurred in rats after the onset of reperfusion in the present study. Weight gain during the experiment was similar among groups (*p* > 0.05). Infarct size, expressed as a percentage of the area at risk, was similar among groups as presented in Fig 1 (F(3,31) = 2.001; *p* > 0.05). No difference was observed for the area at risk between groups and represented approximately 70% of the left ventricle (F(3,31) = 0.528; *p* > 0.05).

### Behavioural tests

**Fig 2:**
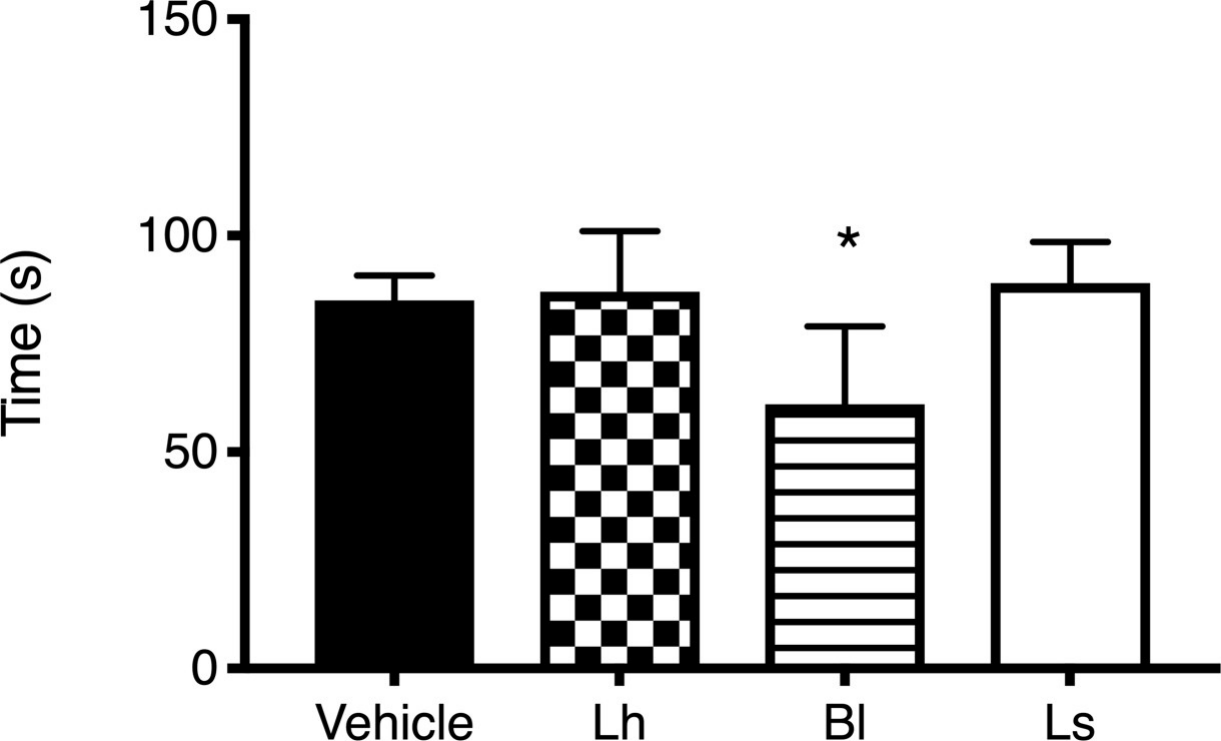
Social interaction. Rats receiving *B*. *longum* (Bl) interacted more with other rats than those receiving vehicle. Rats treated with *Lactobacillus* strains interacted similarly as those receiving the vehicle. Note: *n* = 9–10/group: *p < 0.05 vs. vehicle group: Lh, *L*. *helveticus*; Ls, *L*. *salivarius*.

Analyses of social interaction duration revealed a significant effect between groups (F(3,35) = 8.68; *p* < 0.05; Fig 2). Using the Dunnett test, the post hoc analysis indicated that *B*. *longum* significantly increased the time of interaction between congeners compared to the vehicle group. No difference was observed with the other strains.

**Fig 3:**
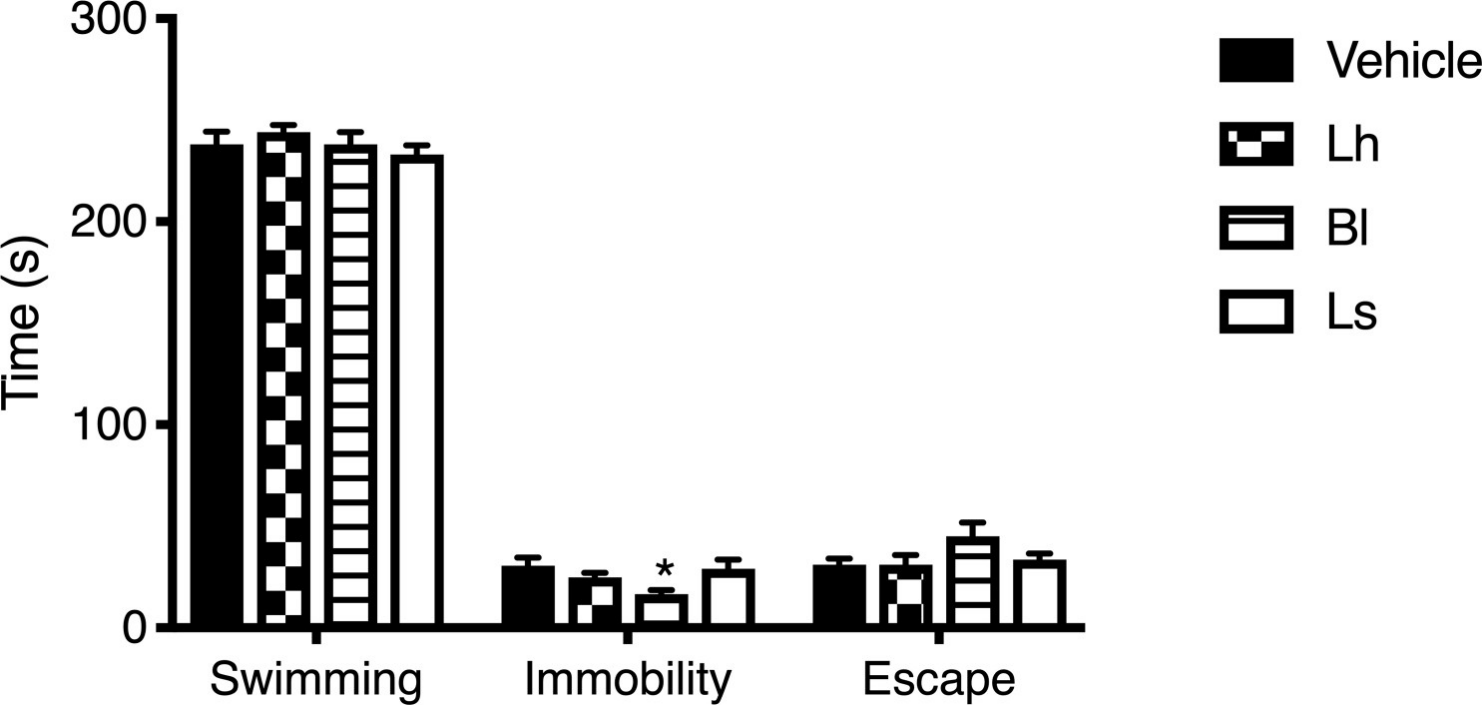
Forced swimming test. MI rats receiving *B*. *longum* (Bl) were less immobile than controls. *Lactobacillus*-supplemented animals presented insignificant differences with the vehicle groups. Note: *n* = 9–11 per group; *p < 0.05 vs. vehicle group; Lh, *L*. *helveticus*; Ls, *L*. *salivarius*.

The results of the forced swim test (Fig 3) showed a significant difference among the groups for the immobility time (F(3,35) = 2.94; *p* < 0.05), but not for the swimming or escape times. Further analyses indicated a significant difference between the *B*. *longum*-and vehicle-supplemented groups. No other differences were detected among the groups.

**Fig 4:**
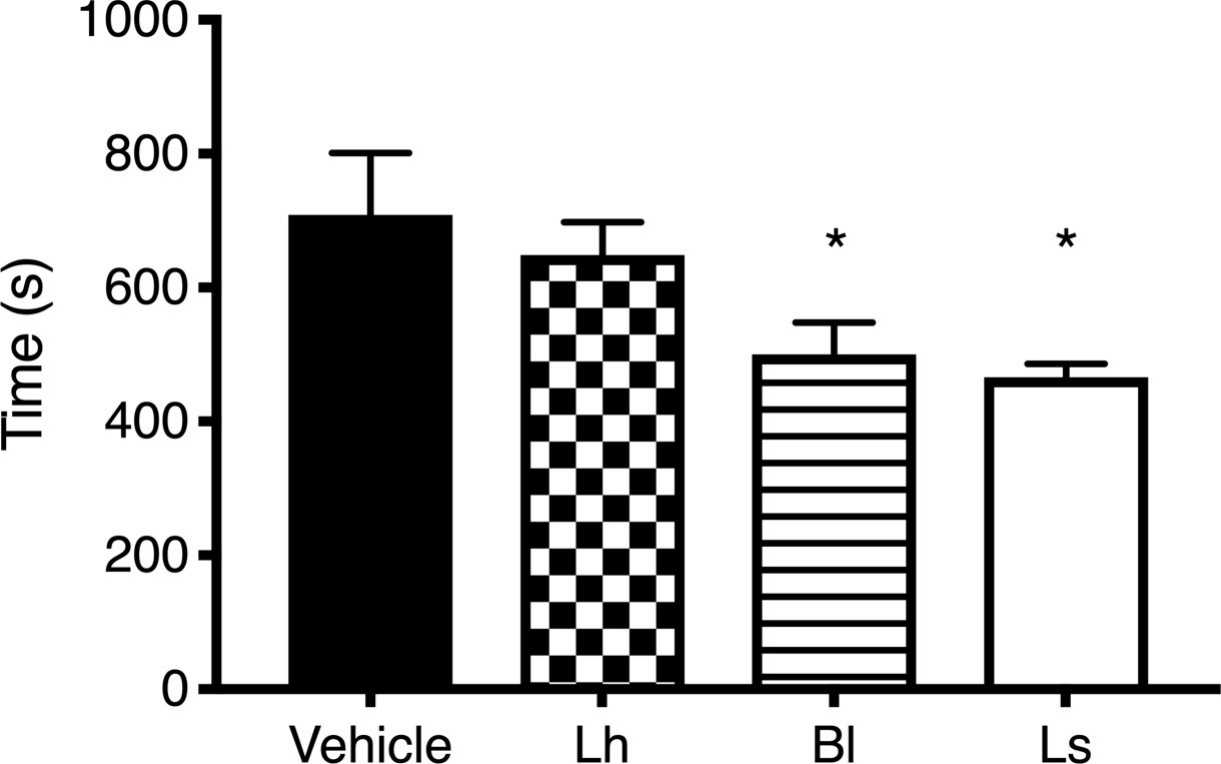
Passive avoidance step-down test. MI rats receiving *B*. *longum* (Bl) or *L*. *salivarius* completed the test faster than vehicle control rats (*p* < 0.05). Animals supplemented with *L*. *helveticus* showed no significant difference with those in the vehicle group. Note: *n* = 8 per group; *p < 0.05 vs. vehicle group; Lh, *L*. *helveticus*; Ls, *L*. *salivarius*.

In the passive avoidance step-down test, results showed that rats receiving *B*. *longum* or *L*. *salivarius* learned the test faster than those in the vehicle group (F(3,15.5) = 4.09; *p* < 0.05). No difference was observed between the *L*. *helveticus* and the vehicle group (Fig 4).

### Caspase-3 activity

**Fig 5:**
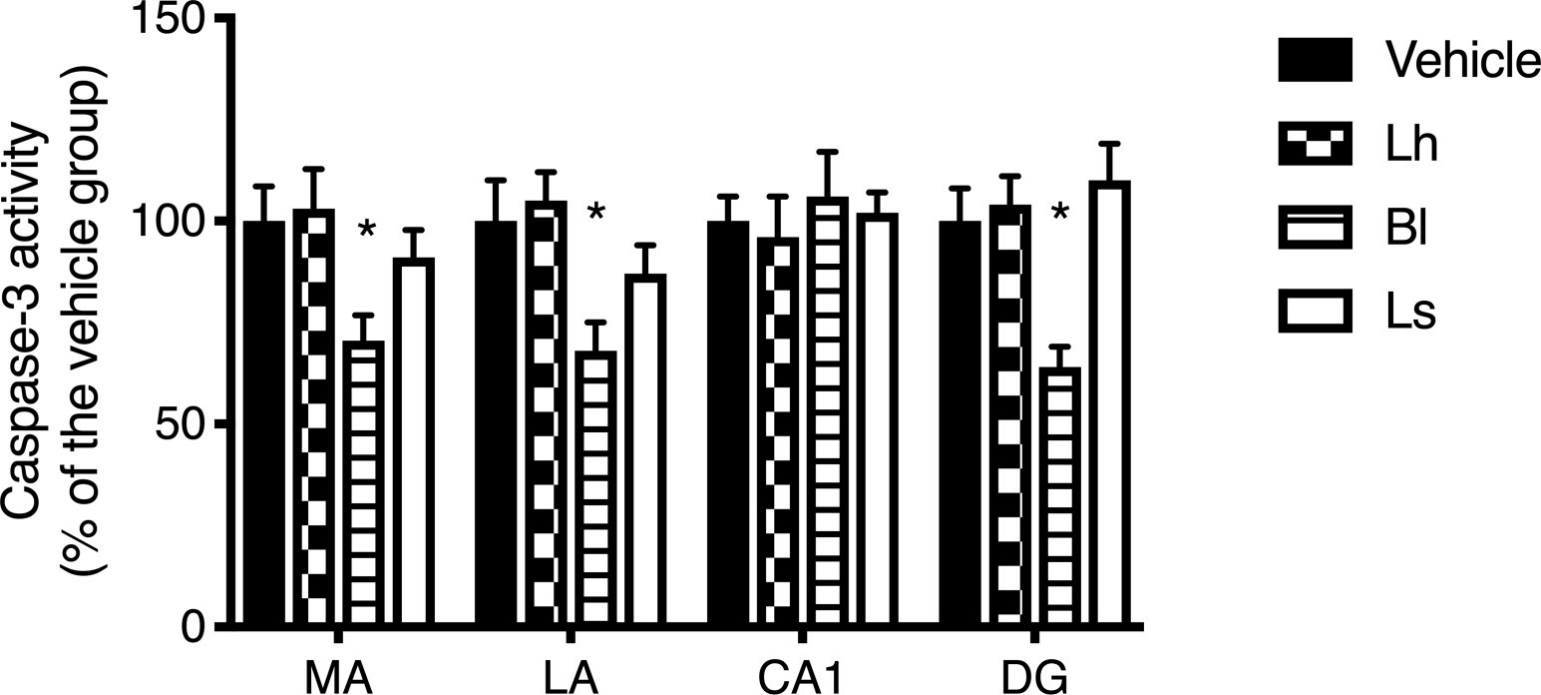
Caspase-3 activity. Caspase-3 activity in the hippocampus (CA1, DG), medial amygdala (MA) and lateral amygdala (LA) of the different groups. Caspase-3 activity was lower in the *B*. *longum* group compared to vehicle; caspase-3 activity was not different between rats receiving either one of the *Lactobacillus* strains or vehicle. Note: *n* = 4–8 per group; *p < 0.05 vs. vehicle group; *Lh*, *L*. *helveticus*; Ls, *L*. *salivarius*.

Three regions showed a significant difference between the groups: medial amygdala (F(3,16) = 3.406, *p* < 0.05), lateral amygdala (F(3,25) = 3.66, *p* < 0.05) and dentate gyrus (F(3,18) = 7.55, *p* < 0.05). For each region, the difference was significant between the *B*. *longum* group and the vehicle (*p* < 0.05), whereas no other differences were detected. For the CA1 region, none of the strains had an effect on caspase-3 activity (Fig 5).

### Plasma CRP concentrations

**Fig 6:**
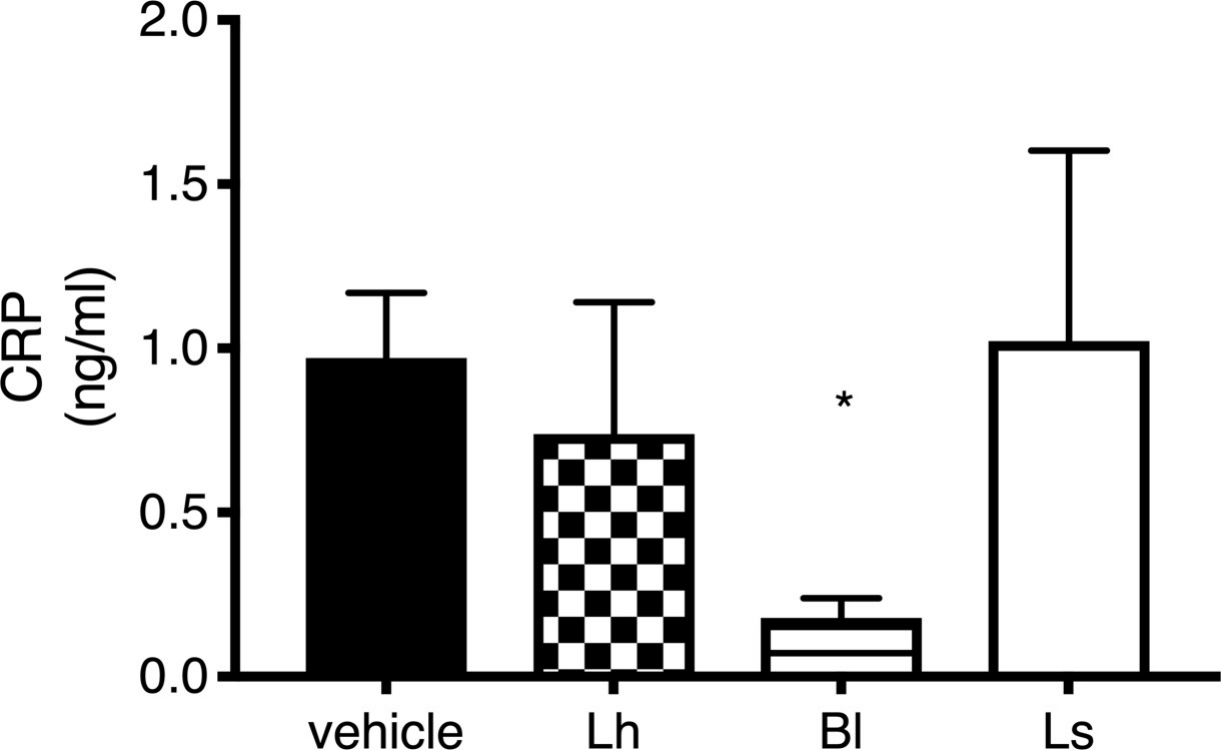
Plasma C-reactive protein (CRP) concentration. Plasma CRP concentration (ng/ml) assessed by ELISA assay after a 24-h reperfusion period (5–6 rats per group). CRP concentrations were significantly lower in the *B*. *longum* group compared to the vehicle group. No differences were observed between groups receiving a *Lactobacillus* strain or vehicle. Note: * *p* < 0.05 vs. vehicle group; *Lh L*. *helveticus*; Ls *L*. *salivarius*.

Since plasma CRP concentrations did not follow a normal distribution, a Kruskal-Wallis test was used. The analysis showed a significant difference between the groups (*p* = 0.016). Pairwise comparisons showed that the animals of the *B*. *longum* group had a lower plasma CRP concentration than the vehicle group. No other difference was observed between the probiotics and control groups (Fig 6).

## Discussion

This study was aimed at assessing the effects of three individual bacterial strains on the behaviour and biochemical changes in the brain after myocardial infarction. In this context, *B*. *longum* mitigated the depressive-like symptoms assessed by three different behaviour tests and reduced caspase-3 activity in the limbic system. Administration of the two *Lactobacillus* strains did not affect these parameters compared to controls, except for *L*. *salivarius* in the passive avoidance step-down test. These effects were independent of the infarct size since no difference was detected between the groups.

Previous studies from our team indicated that the activity of caspase-3, an enzyme involved in apoptosis, was increased in the limbic system, mainly in the hippocampus and amygdala, soon after myocardial infarction [18, 19]. Reduction of apoptosis in the limbic system after MI may have important long-term repercussions on health, although this hypothesis still needs to be tested. It was proposed that neuron loss, which is associated with a reduction of neuronal activity of trophic support [20, 21], could amplify the level of depression or contribute to the pathophysiology of dementia [22].

Our previous data pointed toward an effect of myocardial infarction-induced inflammation [1, 23] underlying the increase in caspase-3 activity [4, 5]. Treatment with molecules harbouring anti-inflammatory properties, such as omega-3 fatty acids[5], the A2A adenosine receptor agonist CGS-21680 [17] or RvD1 [24], has been shown to reduce caspase-3 activity in the limbic region after MI. This hypothesis is supported by the results obtained in the present study since the administration of the *B*. *longum* R0175 reduced caspase-3 activity in some limbic regions in parallel with a reduction in plasma CRP concentrations. These results also indicated that the microbiota may participate in the evolution of the post-MI consequences through modulation of the inflammatory state. On the other hand, we are unable to observe any effect of the two *Lactobacillus* strains that we evaluated although it seems that in specific models some *Lactobacillus* strains may attenuate inflammation [25]. This observation suggests that the mechanism that triggered the inflammation in our model may not be affected by the tested *Lactobacillus* strains. However, in other models of depression where inflammation is not a major issue, *Lactobacillus* strains can be efficient [26] alone or in combination [8].

To document the effect of our different probiotic strains on depressive-like behaviour, we used different tests to obtain converging evidence of a reduction of the depressive behaviour (socialisation, validation of the anti-depression therapy, response to an aversive repeated stimulus)[16]. In accordance with biochemical and physiological analyses, only the *B*. *longum* strain could improve the performance of the rats in these different tests compared to the vehicle group, with one exception; *L*. *salivarius* reduced the time to succeed in the passive avoidance step-down test. These data suggest that *L*. *salivarius* may affect some structures involved in aversive memory and learning other than the amygdala or hippocampus, or by a mechanism that we have not evaluated. This observation warrants further investigation.

Studies reported that interaction between the gut and brain may be possible by humoral, neuronal, immune, and endocrine pathways [27]. One target in our experimental model was the HPA (hypothalamic-pituitary-adrenal) axis, since the dysregulation of the HPA axis participates in the pathophysiology of depression. The dysregulated activation of the glucocorticoid receptors (GR) by agonists induces apoptotic death in neurons [28] whereas anti-depressants increase the expression and function of the GR [29]. It was observed that in the presence of *Bifidobacterium longum* R0175, the GR mRNA levels were elevated in the different regions of the brain in a model of repeated exposure to water exposure avoidance stress, whereas the presence of *L*. *helveticus* did not affect the GR expression [30]. Similarly, the presence of *B*. *longum* reduced the plasma corticosterone levels, whereas the concentration observed in the presence of *L*. *helveticus* did not differ from the positive control. These results suggest that the HPA axis could be considered as a potential target of *B*. *longum* R0175.

Another beneficial effect of *B*. *longum* R0175 demonstrated by the results of this study is its effect on inflammation. Myocardial infarction is an inflammatory pathology, and in the presence of *B*. *longum* R0175, the plasma CRP concentrations were reduced compared to the *Lactobacillus* strains tested. Some studies and meta-analyses demonstrated the link between CRP levels and depression, highlighting the key role of inflammation in depression [31–33].

Our experimental model previously showed an elevation of pro-inflammatory cytokines [5, 7]. This elevation of pro-inflammatory cytokines could impact CRP levels through the activation of different kinases and phosphatases that can lead to the translocation of transcription factors and the increase of CRP protein expression [34].

Interestingly, it was observed that the administration of probiotics resulted in a reduction of the production of pro-inflammatory cytokines [5, 7]. This reduction of pro-inflammatory cytokines by probiotics may result from an effect on the composition of the gut microbiota, the integrity of the intestinal barrier or through the vagus nerve.

Changes in the gut microbiota were reported after MI in rats [35] with an increase of some strains associated with inflammation [36]. Moreover, this change has been associated with intestinal barrier impairment [35]. This data is important since we previously observed in our model that the presence of probiotics had a positive effect on intestinal barrier integrity, which was shown by a reduction in the plasma concentrations of orally administered fluorescein isothiocyanate-dextran (FITC-dextran) [7]. The beneficial effect of *B*. *longum* R0175 in this study could be due to the barrier integrity maintenance and inflammation reduction. Also, some positive effects of the probiotics seem to be related to the activation of the vagus nerve [37], although this theory is not universally accepted [38]. Stimulation of the vagus nerve has been associated with anti-inflammatory properties, which may partly explain the effect of probiotics in post-MI depression [39–43]. These observations are important since some of the effects of the combination of *B*. *longum* R0175 and *L*. *helveticus* R0052 in our post-MI depression model depend on the vagus nerve [15].

Overall, most of the beneficial effects that we previously reported with the combination of *B*. *longum* R0175 and *L*. *helveticus* R0052 in our experimental model of post-myocardial infarction depression are observed using *B*. *longum* R0175 alone, probably via the interference with the inflammatory response.

## Supporting information

S1 Table Infarct size and Area at risk. Infarct size (I) expressed as percent of area at risk(AR) and area at risk expressed as percent of the left ventricle (LV) for each group.
(DOCX)

S2 Table Social interaction. Interactions between animals in seconds
(DOCX)

S3 Table Forced swimming test–Escape time, immobility time and swim time in seconds. (DOCX)

S4 Table Passive avoidance step-down test. Time to succeed the test (in seconds)
(DOCX)

S5 Table Activity of caspase-3 in different regions (% of the control) MA Medial amygdala; LA Lateral amygdala; DG Dentate gyrus. (DOCX)

S6 Table Plasma CRP concentrations. Plasma CRP concentrations (ng/ml) assessed by ELISA assay after a 24-h reperfusion period.
(DOCX)
