# Supplementary material for: Bifidobacterium longum R0175 attenuates post-myocardial infarction depressive-like behaviour in rats
Source: PLoS One. 2019 Apr 22;14(4):e0215101. doi: 10.1371/journal.pone.0215101 (PMC6476493; doi:10.1371/journal.pone.0215101)
Supplement: S1 Table — Infarct size (I) expressed as percent of area at risk(AR) and area at risk expressed as percent of the left ventricle (LV) for each group. (DOCX) [file pone.0215101.s001.docx]

| **Control** | | **Lh** | | **Bl** | | **Ls** | |
| --- | --- | --- | --- | --- | --- | --- | --- |
| **I/AR** | **AR/LV** | **I/AR** | **AR/LV** | **I/AR** | **AR/LV** | **I/AR** | **AR/LV** |
| 23,32 | 68,4 | 37,74 | 68,13 | 39,61 | 71,46 | 47,62 | 68,69 |
| 21,97 | 72,52 | 37,21 | 71,67 | 44,99 | 72,64 | 36,7 | 72,03 |
| 40,08 | 70,55 | 36,7 | 72,03 | 37,67 | 69,62 | 38,69 | 72,89 |
| 48,74 | 80,1 | 35,98 | 70,59 | 33,33 | 72,67 | 42,11 | 70,74 |
| 31,76 | 73,82 | 35,2 | 72,95 | 45,7 | 71,67 | 52,81 | 76,13 |
| 30,38 | 60,08 | 42,86 | 75,25 | 21,4 | 66,39 | 30,58 | 70,12 |
| 30,7 | 71,55 | 25,42 | 64,13 | 44,08 | 58,24 | 49,15 | 72,84 |
| 49,26 | 68,7 | 24,37 | 67,61 | 43,33 | 68,34 | 52,67 | 69,23 |
| 34,72 | 67,71 | 45,22 | 73,71 | 27,56 | 70,75 |  |  |
|  |  |  |  |  |  |  |  |

**S1 Table. Infarct size and Area at risk.** Infarct size (I) expressed as percent of area at risk(AR) and area at risk expressed as percent of the left ventricle (LV) for each group.
