# Supplementary material for: Bifidobacterium longum R0175 attenuates post-myocardial infarction depressive-like behaviour in rats
Source: PLoS One. 2019 Apr 22;14(4):e0215101. doi: 10.1371/journal.pone.0215101 (PMC6476493; doi:10.1371/journal.pone.0215101)
Supplement: S2 Table — Interactions between animals in seconds (DOCX) [file pone.0215101.s002.docx]

| **Control** | **Lh** | **Bl** | **Ls** |
| --- | --- | --- | --- |
| 74 | 93,95 | 255 | 128,23 |
| 88 | 70 | 113,77 | 50,08 |
| 63 | 88 | 125 | 56 |
| 63 | 97 | 224 | 93 |
| 105 | 41 | 114 | 129 |
| 108 | 55 | 123 | 58 |
| 92 | 58 | 143 | 70 |
| 77 | 48 | 204 | 82 |
| 70 | 176 | 148 | 118 |
| 111 | 146 |  | 106 |
|  |  |  |  |

**S2 Table- Social interaction.** Interactions between animals in seconds
