# Supplementary material for: Bifidobacterium longum R0175 attenuates post-myocardial infarction depressive-like behaviour in rats
Source: PLoS One. 2019 Apr 22;14(4):e0215101. doi: 10.1371/journal.pone.0215101 (PMC6476493; doi:10.1371/journal.pone.0215101)
Supplement: S3 Table — (DOCX) [file pone.0215101.s003.docx]

|  | Escape | Immobility | Swim |
| --- | --- | --- | --- |
| Control | 44,89 | 42,56 | 212,55 |
|  | 17,49 | 16,49 | 266,02 |
|  | 7,79 | 16,74 | 275,47 |
|  | 31,27 | 17,07 | 251,66 |
|  | 33 | 33,85 | 233,15 |
|  | 34,25 | 24,32 | 241,43 |
|  | 38,75 | 55,46 | 205,79 |
|  | 41,43 | 21,44 | 237,13 |
|  | 31,64 | 29,16 | 239,2 |
|  | 29,99 | 49,18 | 220,83 |
|  | 31,05 | 30,627 | 238,323 |
| Lh | 41,65 | 23,19 | 235,16 |
|  | 9,51 | 41,15 | 249,34 |
|  | 47,09 | 28,97 | 223,94 |
|  | 11,12 | 29,18 | 259,7 |
|  | 61,82 | 11,41 | 226,77 |
|  | 25,4 | 23,18 | 251,42 |
|  | 33,38 | 26,73 | 239,89 |
|  | 22,73 | 18,85 | 258,42 |
|  | 30,14 | 26,31 | 243,55 |
|  | 28,46 | 20,03 | 251,51 |
| Bl | 43,91 | 11,02 | 245,07 |
|  | 60,04 | 8,13 | 231,83 |
|  | 100,48 | 12,47 | 187,05 |
|  | 40,36 | 8,88 | 250,76 |
|  | 57,95 | 16,03 | 226,02 |
|  | 37,15 | 21,16 | 241,69 |
|  | 10,74 | 28,3 | 260,96 |
|  | 28,84 | 21,86 | 249,3 |
|  | 32,16 | 18,29 | 249,55 |
|  | 38,26 | 19,64 | 242,1 |
| Ls | 38,03 | 8,5 | 253,47 |
|  | 34,06 | 31 | 234,94 |
|  | 24,88 | 61,32 | 213,8 |
|  | 53,44 | 11,5 | 235,06 |
|  | 42,86 | 42,64 | 214,5 |
|  | 22,82 | 28,95 | 248,23 |
|  | 21,93 | 24,64 | 253,43 |
|  | 32,78 | 27,41 | 239,81 |
|  | 31,28 | 24,74 | 243,98 |

**S3 Table.** **Forced swimming test –** Escape time, immobility time and swim time in seconds
