# Supplementary material for: Bifidobacterium longum R0175 attenuates post-myocardial infarction depressive-like behaviour in rats
Source: PLoS One. 2019 Apr 22;14(4):e0215101. doi: 10.1371/journal.pone.0215101 (PMC6476493; doi:10.1371/journal.pone.0215101)
Supplement: S4 Table — Time to succeed the test (in seconds) (DOCX) [file pone.0215101.s004.docx]

| **Control** | **Lh** | **Bl** | **Ls** |
| --- | --- | --- | --- |
| 733 | 707,52 | 760,53 | 496,28 |
| 796 | 792 | 653 | 397 |
| 466 | 611 | 487 | 442 |
| 501 | 875 | 429 | 559 |
| 858 | 583 | 375 | 522 |
| 624 | 491 | 399 | 402 |
| 459 | 655 | 420 | 398 |
| 1234 | 480 | 478 | 481 |

S4 Table. **Passive avoidance step-down test.** Time to succeed the test (in seconds)
