# Supplementary material for: Bifidobacterium longum R0175 attenuates post-myocardial infarction depressive-like behaviour in rats
Source: PLoS One. 2019 Apr 22;14(4):e0215101. doi: 10.1371/journal.pone.0215101 (PMC6476493; doi:10.1371/journal.pone.0215101)
Supplement: S5 Table — (DOCX) [file pone.0215101.s005.docx]

|  | Control | Lh | Bl | Ls |
| --- | --- | --- | --- | --- |
| MA | 74,5 | 106,8 | 53,3 | 101,5 |
|  | 125,5 | 98,5 | 74,5 | 73,6 |
|  | 109,4 | 127,2 | 64,2 | 87,2 |
|  | 90,6 | 80 | 91,2 | 118,3 |
|  | 100 |  | 70 | 89,4 |
|  |  |  |  | 76,4 |
| LA | 100,8 | 72,8 | 92,1 | 84,2 |
|  | 99,2 | 99,2 | 62,9 | 136 |
|  | 109,8 | 104,6 | 90,4 | 91,5 |
|  | 90,2 | 106,9 | 37,2 | 65 |
|  | 83,9 | 132,6 | 56,7 | 63,8 |
|  | 116,1 | 102,9 |  | 81,6 |
|  | 75,2 | 117,3 |  |  |
|  | 124,8 |  |  |  |
| Ca1 | 100 | 96,9 | 97,2 | 136,4 |
|  | 100 | 74,8 | 118,4 | 79,6 |
|  | 127 | 105,7 | 80,2 | 92,2 |
|  | 73 | 107 | 130,6 | 124,5 |
|  |  |  |  | 75 |
| DG | 108,6 | 115 | 49,3 | 123,9 |
|  | 91,4 | 103 | 70 | 131,5 |
|  | 127,7 | 94 | 62,6 | 96,4 |
|  | 72,3 | 108,8 | 62,9 | 114,3 |
|  | 106,4 | 126,8 | 76,3 | 83,5 |
|  | 93,6 | 77,9 |  |  |

**S5 Table- Activity of caspase-3 in different regions** (% of the control) MA Medial amygdala; LA Lateral amygdala; DG Dentate gyrus.
