# Supplementary material for: Bifidobacterium longum R0175 attenuates post-myocardial infarction depressive-like behaviour in rats
Source: PLoS One. 2019 Apr 22;14(4):e0215101. doi: 10.1371/journal.pone.0215101 (PMC6476493; doi:10.1371/journal.pone.0215101)
Supplement: S6 Table — Plasma CRP concentrations (ng/ml) assessed by ELISA assay after a 24-h reperfusion period. (DOCX) [file pone.0215101.s006.docx]

| **Control** | **Lh** | **Bl** | **Ls** |
| --- | --- | --- | --- |
| 1,32 | 0,412 | 0,37 | 3,815 |
| 0,995 | 0,295 | 0,363 | 1,229 |
| 1,365 | 1,81 | 0,162 | 0,23 |
| 0,562 | 0,427 | 0,134 | 0,379 |
| 0,62 | 0,75 | 0,032 | 0,37 |
|  |  | 0,018 | 0,117 |
|  |  |  |  |

**S6 Table. Plasma CRP concentrations.** Plasma CRP concentrations (ng/ml) assessed by ELISA assay after a 24-h reperfusion period.
